# Health risk assessment via Monte Carlo simulation and sensitivity analysis for fluoride and nitrate content in bottled waters consumed in Kermanshah city, Iran

**DOI:** 10.1038/s41598-025-89439-6

**Published:** 2025-02-11

**Authors:** Hanieh Yari Mianeh, Laya Amiri, Ali Jafari, Nasim Nourozi

**Affiliations:** 1 Department of Environmental Health Engineering, School of Health, Kermanshah University of Medical Sciences, Kermanshah, Iran; 2 Student Research Committee, Kermanshah University of Medical Sciences, Kermanshah, Iran; 3 Health, Safety and Environment Technologies Research Core, Health Technology Institute, Kermanshah University of Medical Sciences, Kermanshah, Iran

**Keywords:** Bottled water, Health risk assessment, Fluoride, Nitrate, Non-carcinogenic, Monte Carlo, Environmental sciences, Risk factors

## Abstract

Bottled water consumption has increased in recent decade due to many reasons, especially significant decline in water quality and quantity. The concentration of fluoride and nitrate in bottled waters may vary based on brands and locations. This study was carried out to determine the levels of fluoride and nitrate in bottled waters consumed in Kermanshah city and assess the related non -carcinogenic risks. Totally, 22 brands of bottled water were collected from markets. Fluoride and nitrate measurement was conducted via a UV-visible spectrophotometer (DR-5000). From the results, Fluoride and nitrate levels in the studied bottled waters were 0.32 ± 0.18 mg/L and 2.3 ± 1.41 mg/L, respectively. The risk of non-carcinogenic in term of HQ for fluoride exposure, for only 2 brands of bottled water were > 1 for infants group. HQ was less than 1 for nitrate in all the brands for all the age groups revealed non-carcinogen risks. Hazard index (HI) calculation showed that only in 2 brands of bottled water HI was > 1 for infants group. The HI were as infants (0.64) > children (0.36) > teenagers (0.27) > adults (0.24). From Monte Carlo simulation, 95th Percentile for nitrate and fluoride was less than 1 for all the groups. This result indicated non-carcinogenic risks of nitrate and fluoride for 95% of the studied groups. Moreover, sensitivity analysis showered that concentration for both nitrate and fluoride had the highest effect on HQ for all the groups. From this work, although fluoride and nitrate content in the bottled waters were at standard range, but infants were proportionally at higher risk.

## Introduction

Safe water is defined as water free of any pollutants (chemical, biological and radioactive agents), and with no adverse health effects through long-term consumption. Accordingly, adequate, safe, and accessible drinking water must be supplied for all. Many pollutants, including inorganic, organics and heavy metals may exist in waters and induce human risks^1^.Through some environmental control measures water quality can preserved from the source^2^. Among the various inorganics considered in drinking water quality, fluoride and nitrate are of noticeable elements due to their assigned adverse human health effects.

Fluoride is an essential element for human at recommended values. It can effectively reduces dental caries without harmful effects at allowable levels, but, at extra levels it can cause serious dental and sclerosis disorders^3^. Fluoride also may cause adverse effects on brain, decrease intelligence quotient (IQ) and increase blood hypertension^4,5^. However world health organization recommended a guideline vale of 0.5–1.5 of fluoride in drinking waters^3^.

Fluoride may occur naturally in waters or it may be added to drinking water in controlled amounts in some areas. Fluoride can be found in drinking waters, foods^6–11^. Ingestion is considered as the main source of intake through drinking^12^. Many studies have reported different levels of fluoride in drinking waters and the related health effects around the world. In some regions, fluoride content has been reported as high as 2800 mg/L and consequent related obvious skeletal fluorosis^13–22^. Such waters should be defluoridated using proper methods^23,24^.

Nitrate is also a harmful compound that can enter the body through water consumption. Although other sources of nitrate exposure are considered but drinking, water is mainly considered for different groups of population. Nitrate through a process can disorder in blood red cell function and produce methhemoglobinemia (metHb) and resulting in blue baby especially in children^3^. Other adverse possible risks related to nitrate intake have been reported in some parts due to elevated nitrate intake especially through drinking waters^25^. Nitrate in drinking water can cause many problems, while some cancers are also possible^3,26^.

In recent decade, bottled water consumption has dramatically increased worldwide due to many reasons. Bottled water has been considered as the main source of drinking water supply in some arid and semiarid countries. Bottled water consumption has attracted due to better taste and higher clearness. It is estimated that global growth rate of bottled water consumption is about 7%. About 89 billion liter bottled drinking water consumed in 2006 ^27^. Although the main consumers are currently European, consumption rates have also increased in Asia, and the Pacific is increasing faster, by approximately 15% ^28^. In areas where drinking water of good quality is not available and water purification facilities are not available, the probability of bottled water use is higher. Meanwhile, the United States and European countries account for more than 35% of the world’s bottled water market (14). This amount of bottled water can cause problems if there is no monitoring of their quality and the amount of some substances in them is higher. Safe drinking water supply is the primary and essential community public health concern. For this, water quality assessment is usual and necessary. Accordingly, many studies have been conducted to investigate the quality of drinking waters. In various studies, biological factors, micro plastics and chemical have been investigated^29–32^. Different works have also been performed for nitrate and fluoride content in bottled waters different areas mainly in standard range^8,33–39^. As bottled water are different origins and sources and may some treatment processes may be conducted on them so their quality may differ from one brand to another bottled treated drinking waters and mineral bottled water are in some places are categorized and sold with different qualities.

Human health risk assessment (HRA) serves as a key tool in public health management and can help enhance the quality of life and public health. HRA is a helpful methodology used to estimate the possible and elevated of adverse effects caused by hazardous parameters in human health. Furthermore, HRA contain the scientific experiments and assessment of data^12,40^. In addition, HRA has been widely employed in many studies to evaluate health risk of various agents and bottled waters quality and possible health risks^29,30,34,36–38,41–43^.

Bottled water consumption has increased in Kermanshah city in recent years. In public meeting and large ceremonies, people mainly used bottled water. In some parts of the city people regularly used bottled water due to low water quality, lack of water and other health reasons. Regarding the importance of drinking water quality monitoring is necessary as an important way for receiving fluoride and nitrate, evaluation of fluoride content in water resources. Accordingly, this study was conducted to evaluate fluoride and nitrate content is bottled waters consumed in Kermanshah and assesses the related risk health among various age groups (namely; children, teenagers, and adults).

## Materials and methods

### Samples collection and study area

**Fig. 1:**
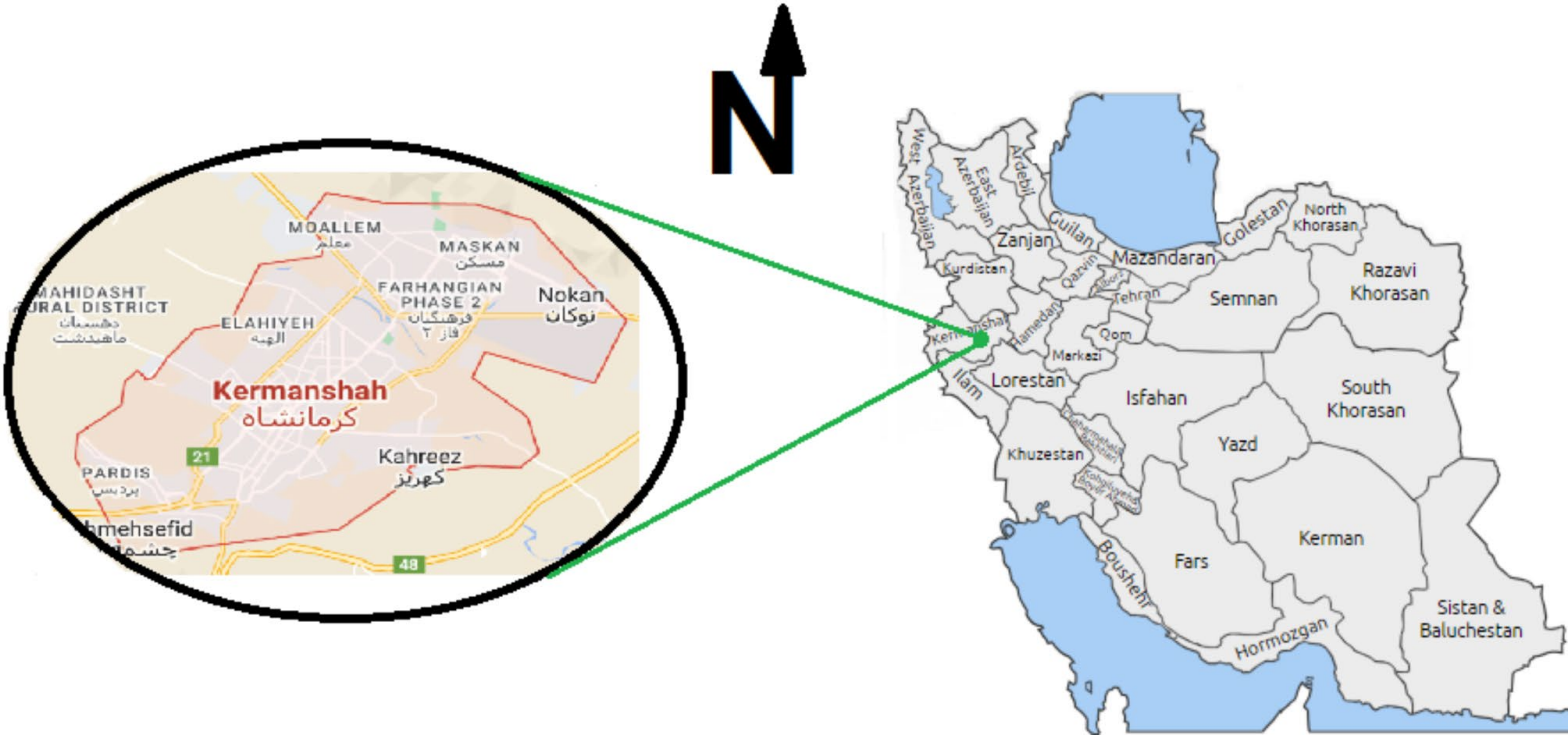
Kermanshah city location in Iran.

Kermanshah city is located in west of Iran (Fig. 1), with a population of about 1,000,000 people and in mountainous area. The main sources of water supply are ground waters and dam reservoirs. Nevertheless, in last decades with an increase in city development the water demand has increased due to some reasons. The summers are nearly warm with annual precipitation of 456.8 mm and average temperature of 14 ^o^C. In this work 22 brands of mostly consumed bottled water, (treated bottled water, and minerals bottled water), were collected from the main markets throughout the city. The samples were collected in triplicate for accurate results. The samples were analyzed for fluoride and nitrate content and the averages of the measurements were reported and used for HRA.

The samples were measured for both fluoride and nitrate using spectrophotometric methods as follows. Nitrate concentration was determined via DR 5000 spectrophotometer (Hach, Germany) at wavelengths of 220 nm. To remove the possibility of interference of organic substances in the method, a correction was made at wavelength of 275 nm according to standard methods^44^. In addition, HCl (1 N) used to remove the interference of alkalinity in the samples. SPANDS method was used to determine fluoride level in the samples at 507 nm using DR 5000 spectrophotometer^44^. The results of fluoride and nitrate concentration were illustrated using Microsoft Excel.

### Health risk assessment

Concerns relating to drinking water contaminated with fluoride and nitrate is increasing in Iranian pupation due to elevated public awareness. HRA is a known method to assess the possible adverse effects on human health over a specific period of time [33]. Due to different physiological functions of the population, the target groups were categorized into 4 age groups. In this study HRA used as a scientific and useful method to document and answer the public concerns. For this, HRA was performed for 4 groups of the different exposed people, namely infants (< 2 years), children (> 2 and < 6 years), adolescents (> 6 and < 16 years) and adults (> 16 years)^45,46^. The daily consumption of nitrate and fluoride were calculated following Eqs. 2^9^.

1 $$\:\text{H}\text{Q}=\:\frac{\text{E}\text{D}\text{I}}{\text{R}\text{f}\text{D}}$$

2 $$\:EDI=\frac{C\times\:\text{I}\text{R}\times\:\text{E}\text{F}\times\:\text{E}\text{D}}{\text{B}\text{W}\times\:\text{A}\text{T}}$$

The reference dose (RfD) for an individual contaminant is expressed in mg/kg of body weight (BW) per day. RfD is an important factor in HRA. In present work, RfD value for nitrate and fluoride were 1.6 (mg/kg/day) and 0.06, respectively^48^. The parameters definitions, unit, and values ​​for HQ calculation for different groups are shown in Table 1.

From the table (Table 1), EDI is daily intake of nitrate and fluoride (mg/kg), C is nitrate and fluoride concentration in potable bottled water (mg/L), IR is the daily consumption of drinking water, EF is exposure frequency, AT is average exposure time and BW is body weight (kg) for each exposed group.

**Table 1:** Various variables and values for Monte Carlo simulation and uncertainty analysis of nitrate and fluoride.

	Parameter	Fluoride & Nitrate Concentration	Ingestion Rate	Exposure Frequency	Exposure Duration	Body Weight	Averaging time	Reference dose- Fluoride	Reference dose- Nitrate
	Symbol	C	IR	EF	ED	BW	AT	RfD-F	RfD-NO3
	Unit	mg/L	L/day	day	Year	kg	day	mg/kg.day	mg/kg.day
**Groups**	**Distribution**	lognormal	lognormal	-	-	Normal	-	-	-
	**Infants**	-	0.7	365	1.5	7.5	547.5	0.06	1.6
	**Children**	-	0.8	365	4	15	1460	0.06	1.6
	**Teenagers**	-	2.0	365	13	50	4745	0.06	1.6
	**Adults**	-	2.5	365	40	72	14,600	0.06	1.6

### Monte Carlo simulation and sensitivity analysis

Monte Carlo Simulation as a known computer-driven approach, applies repeatedly random sampling and analytical methods to obtain a probabilistic estimation for calculating outcome models^49^. Generally, risk assessments contain some levels of uncertainties needs to be considered to obtain consequent risk management action^48,49^. The Monte Carlo Simulation method combines an extensive array of model and parameter assumptions to predict the overall probability distribution of exposure variables, moving beyond mere upper-bound single-point estimates or specific values. This approach results in a robust risk distribution outcome^49^.

To minimize the uncertainty level, Monte Carlo simulation has been used to achieve an acceptable risk assessment. Therefore, wide variety of variables is commonly applied in place of a single-point value thereby allowing the estimation process to be conducted with multiple levels of reliability^49^.

Nitrate and fluoride contents (C), duration of exposure(ED), body weight (BW), and intake rate (IR) were applied to calculate the potential distribution or uncertainty^50^. For this, a large range of the values with a large random trails (10000) were applied for a range of confidence (1 to 99%)^50^. Thus, the modifications in incidence rates within this study were taken into account for each population, which helps to reduce uncertainty and enhance the accuracy of health risk outcomes.

These probability distributions are used as the input distributions for exposure model parameters. During a single trial, values are randomly selected from the defined possibilities (the range and shape of the distribution) for each uncertain variable and then the output of the model is calculated^49^. If a simulation run for 10,000 trials 10, 000 forecasts (or possible outcomes) are created compared to the single outcome obtained in the deterministic approach^50,51^. The probability distribution resulted from exposure to each compound is subsequently utilized to assess the Hazard Quotient (HQ) and consequently hazard index (HI) values.

### Sensitivity analysis

Sensitivity analysis is commonly performed to determine which variable significantly affects the results of risk assessment. In other words, Sensitivity analysis examines how variations in the input variables of a mathematical model influence the outputs, finally establishing a relationship between input parameters and output variations^49^.In this study, Crystal Ball (version 11.1.2.4, Oracle, Inc., USA) for Monte Carlo simulation and sensitivity analysis via 10,000 repetition^46,50,51^. The parameters for sensitivity analysis as applied for the Monte Carlo Simulation are outlined in Table 1 the probability distribution for functions applied in the Monte Carlo simulation and sensitivity analysis were acquired as applied in other works^46,51,52^.

###  Hazard index

The Hazard Index (HI) is applied by the Environmental Protection Agency (EPA), particularly within the Superfund program, to assess health risks addition linked to mixture of component and as a screening-level check for potential health risks^53,54^. The HI is determined by adding hazard quotients (HQs) for the individual component in the mixture^50,55^. A HQ is the ratio of a chemicals exposure level to related reference dose, Eq. 1.

In this work, health risk effects related to sum of nitrate and fluoride hazard was calculated in term of HI for four age groups, namely infants, children, teenagers, and adults using Eq. (3) ^54^.

3 $${\rm HI}\,=\,{\rm HQ}_{\rm Nitrate} +{\rm HQ} _{\rm Fluoride}$$

## Results and discussion

### Fluoride content

**Fig. 2:**
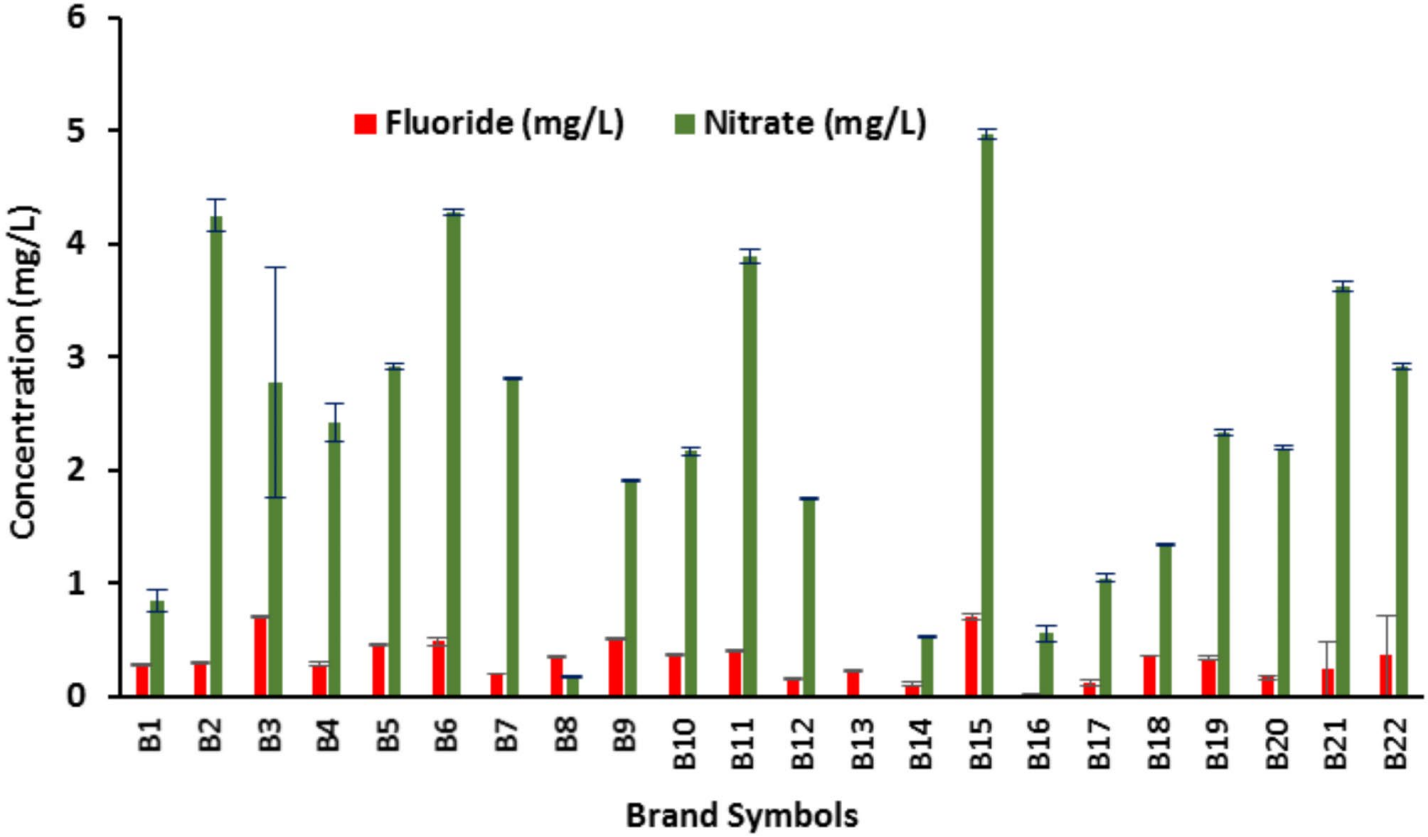
Fluoride and nitrate concentration in different bottled water brands.

As shown in Table 2, fluoride concentrations ranged from 0.01 mg/L to 0.71 mg/L (0.32 ± 0.18). According to the results shown in Fig. 2, the maximum concentrations of fluoride were assigned to B3 and B15. Generally, 3 out of the 22 brands were within the standard range recommend by WHO (0.5–1.5 mg/L) and about 86% of the samples are less than minimum value (0.5 mg/L) suggested by WHO. 8 samples have not labeled the fluoride concentration although the measured fluoride content is lower than standards. More inspection and control on the labeled list values are important by official organization for further assessment. 8 of the collected samples were mineral waters (MW) and 16 samples were drinking bottled water (DW). MW are mainly take from ground waters (e.g. springs) and bottled without further purification. However, drinking waters are treated water via purifications methods. Many other works conducted on bottled water quality for fluoride reveled the same levels of fluoride^31,32,35,36,51,57,58^. Nevertheless, the levels for fluoride in non-bottled waters are varies in, higher levels than WHO guideline, up to 10 mg/L, have also been reported in Iran^59,60^.

#### Fluoride health risk assessment

Health risk assessment (HRA) of fluoride in bottled waters consumed in Kermanshah was assessed using HQ as a non-carcinogenic risk approach. Levels of fluoride exposure were calculated for the four group categories. The average of EDI values for infants, children, teenagers and adults are 0.03 (mg/kg.day), 0.02(mg/kg.day), 0.01 (mg/kg.day), and 0.01 (mg/kg.day), respectively.

From the results, infants and children showed higher level of EDI compared to teenagers and adults. Therefore, these groups are supposed to be at higher risk. The same finding was previously reported^54^.

From USEPA, exposure of children to fluoride at values of higher than reference dose (0.06 mg/kg.bw day) may cause dental fluorosis^48^. From the results, two brands (B3 and B15) can cause higher EDI than 0.06 and increase the risk of dental fluorosis in infants. It has been reported that, at estimated exposure dose of 0.3 mg F kg − 1 bw the reveled primarily health signs^61^. In this work, none of the target groups exposed this value. The hazard quotient (HQ) for fluoride exposure was calculated for four age groups. The HQ values beyond 1 signify that the population in the studied area is exposed to higher risk levels of developing non-carcinogenic disorders. As shown in Table 2, HQ values related to 2 brands (B3 and B15) were higher than 1 for infants. HQ assigned to other brands for all groups were less than 1, such the values less than 1 are not of potential concern. The HRA of fluoride for exposed target groups was ordered as infants > Children > Teenagers > Adults. Based on the obtained data, infants and children are more likely to suffer from adverse health effects associated with the consumption of fluoride-content water. In an high consistency, it has been reported that children risk to fluoride exposure id higher in children group^62^. It has been clarified that Infants and children with lower body weight is the main affecting factor for this as previously mentioned for Fluoride^29,62,63^. Based on the calculated HQs the result of present work showed that, the risk of dental fluorosis for the all the age groups are not considerable. It should be noticed that the results are based on bottled water consumption, but overall, as the fluoride intake is happened through other main sources like distribution drinking water sources, foods, beverages, tea meat and fluoride supplements so on the cumulative risk can be higher and the risk can increase the risk. Importantly, the acceptable level of fluoride in drinking water should be mentioned with including other intake sources.

### Nitrate content

As shown in Table 2, Nitrate concentrations ranged from 0 to 5 mg/L (2.3 ± 1.14). According to the results shown in Fig. 2, the maximum concentration of nitrate was assigned to B5. None of the samples exceeded the guideline value recommend by WHO (45 mg/L). 3 samples have not labeled the nitrate content although the measured nitrate content is lower than WHO guideline and Iranian standard (50 mg/L). through the studies review, it has been reported that nitrate content was higher than that labeled in many cases, and not in many cases not labeled in Iranian bottled water^64^.More inspection and control on the labeled list values are necessary by officials for further assessment. As mentioned previously, 8 of the collected samples were mineral waters (MW) and 16 samples were drinking bottled water (DW). The mineral water are mainly take from ground waters (e.g. springs) and put in a bottle without further purification. Nevertheless, drinking bottled waters are treated via purifications methods. As the main sources of water and purification process are not clarified by the factories, it is difficult to speak about the efficiencies and possible default through the water processing. Many other works conducted on bottled water quality for nitrate reveled the levels less than Iranian standard of nitrate in many samples^33,34,38,39,51,65^. However, the ranges for nitrate contents in non-bottled waters vary with high levels^26,64^.

#### Nitrate health risk assessment

Health risk assessment (HRA) of nitrate in bottled waters consumed in Kermanshah was assessed using HQ as a non-carcinogenic risk approach. Levels of nitrate exposure were calculated for the four categories. The average of EDI values for infants, children, teenagers and adults ere 0.21 (mg/kg.day), 012(mg/kg.day), 0.09 (mg/kg.day), and 0.08 (mg/kg.day), respectively.

From the results, infants and children showed higher level of EDI compared to teenagers and adults. Therefore, these groups are supposed to be at higher risk. However, this vales compare to other sources intake is low. For instance, Rezaei et al., reported higher EDI (1.14 mg/kg.day) for children than adults in water distribution^30^. Qasemi et al., reveled the same order (infants > children > adults)^54^.

The hazard quotient (HQ) for nitrate exposure was calculated for four age groups. The HQ values exceed 1 clarify that the exposed groups to higher health risk levels of non-carcinogenic effects. Table 3 presents the results of HQ related to Fluoride, nitrate for different age groups. From the results, the average of HQ values for infants, children, teenagers, and adults ere 0.13, 0.07, 0.06, and 0.05, respectively. From this study, HQ values related to nitrate were not higher than 1 for the groups. Generally the HRA of nitrate for exposed target groups was ordered as infants > Children > Teenagers > Adults for the collected samples. Although HQ for all the group ages is less than 1 (HQ < 1) indicates that no adverse health effects. From the results, infants and children revealed higher HQ compared to teenagers and adults. Therefore, these groups (infants and children) are comparably at higher risk. However, HQ values compare to other sources intake are low. For instance, Rezaei et al., revealed mean of HQ for infants > children > teenagers > adults in public water distribution^30^. Based on the obtained data, infants and children are more likely to suffer from adverse health effects associated with the consumption of nitrate-content water It has been clarified that Infants and children with lower body weight is the main affecting factor for this as previously mentioned for nitrate reference^29,51,62,63^.

As noted previously it should be mentioned that the results are from bottled water consumption, but as the nitrate is ingested from other main source like public water distribution systems, wells, beverages and so on, the cumulative related health risk could be higher.

**Table 2:** Fluoride and nitrate levels and the labeled value in different bottled waters.

Symbol	Type	Fluoride			Nitrate		
		Labeled value (mg/L)	Measured(mg/L)	±SD	Label value (mg/L)	Measured(mg/L)	±SD
B1	DW*	0.7	0.28	0.01	1.3	0.85	0.10
B2	MW**	NL***	0.30	0.01	1.9	4.25	0.14
B3	MW	NL	0.71	0.01	2	2.78	1.02
B4	DW	0.1	0.29	0.01	0.4	2.42	0.17
B5	MW	0.15	0.45	0.01	8	2.91	0.03
B6	DW	NL	0.49	0.03	NL	4.28	0.03
B7	MW	0.09	0.20	0.00	1.1	2.81	0.01
B8	MW	0.414	0.35	0.01	0.74	0.18	0.01
B9	MW	NL	0.51	0.01	4.5	1.91	0.01
B10	DW	0.5	0.37	0.01	< 2	2.17	0.03
B11	DW	0.01	0.41	0.01	9.5	3.89	0.06
B12	MW	< 0.005	0.16	0.01	< 0.8	1.75	0.01
B13	DW	NL	0.22	0.00	< 1	0.02	0.01
B14	MW	0.1	0.11	0.01	2.45	0.53	0.01
B15	DW	0.25	0.70	0.02	2.56	4.96	0.04
B16	DW	< 1	0.01	0.01	< 10	0.56	0.07
B17	DW	0.07	0.11	0.03	0.61	1.04	0.04
B18	DW	0.05	0.36	0.00	6.8	1.33	0.01
B19	DW	NL	0.34	0.01	NL	2.33	0.03
B20	DW	NL	0.16	0.02	NL	2.20	0.01
B21	DW	0.22	0.24	0.24	7.22	3.63	0.04
B22	DW	NL	0.36	0.35	5	2.91	0.03

*Drinking water, **Mineral water, ***Not Labeled.

**Table 3:** HQ Fluoride, nitrate for different age groups.

HQ-Fluoride				HQ-Nitrate			
Infants	Children	Teenage	Adults	Infants	Children	Teenage	Adults
0.44	0.25	0.19	0.16	0.25	0.14	0.11	0.09
0.46	0.26	0.20	0.17	0.16	0.09	0.07	0.06
1.11	0.62	0.47	0.41	0.14	0.08	0.06	0.05
0.45	0.25	0.19	0.17	0.17	0.09	0.07	0.06
0.70	0.39	0.30	0.26	0.25	0.14	0.11	0.09
0.76	0.42	0.33	0.28	0.16	0.09	0.07	0.06
0.31	0.17	0.13	0.12	0.01	0.01	0.00	0.00
0.55	0.30	0.23	0.20	0.11	0.06	0.05	0.04
0.80	0.45	0.34	0.30	0.13	0.07	0.05	0.05
0.58	0.32	0.25	0.22	0.23	0.13	0.10	0.08
0.63	0.35	0.27	0.24	0.10	0.06	0.04	0.04
0.25	0.14	0.11	0.09	0.00	0.00	0.00	0.00
0.35	0.19	0.15	0.13	0.03	0.02	0.01	0.01
0.17	0.09	0.07	0.06	0.29	0.16	0.12	0.11
1.09	0.61	0.47	0.41	0.03	0.02	0.01	0.01
0.02	0.01	0.01	0.01	0.06	0.03	0.03	0.02
0.17	0.10	0.07	0.06	0.08	0.04	0.03	0.03
0.55	0.31	0.24	0.21	0.14	0.08	0.06	0.05
0.53	0.29	0.23	0.20	0.13	0.07	0.05	0.05
0.26	0.14	0.11	0.10	0.21	0.12	0.09	0.08
0.38	0.21	0.16	0.14	0.17	0.09	0.07	0.06
0.56	0.31	0.24	0.21	0.13	0.07	0.06	0.05

### Hazard index (HI)

**Fig. 3:**
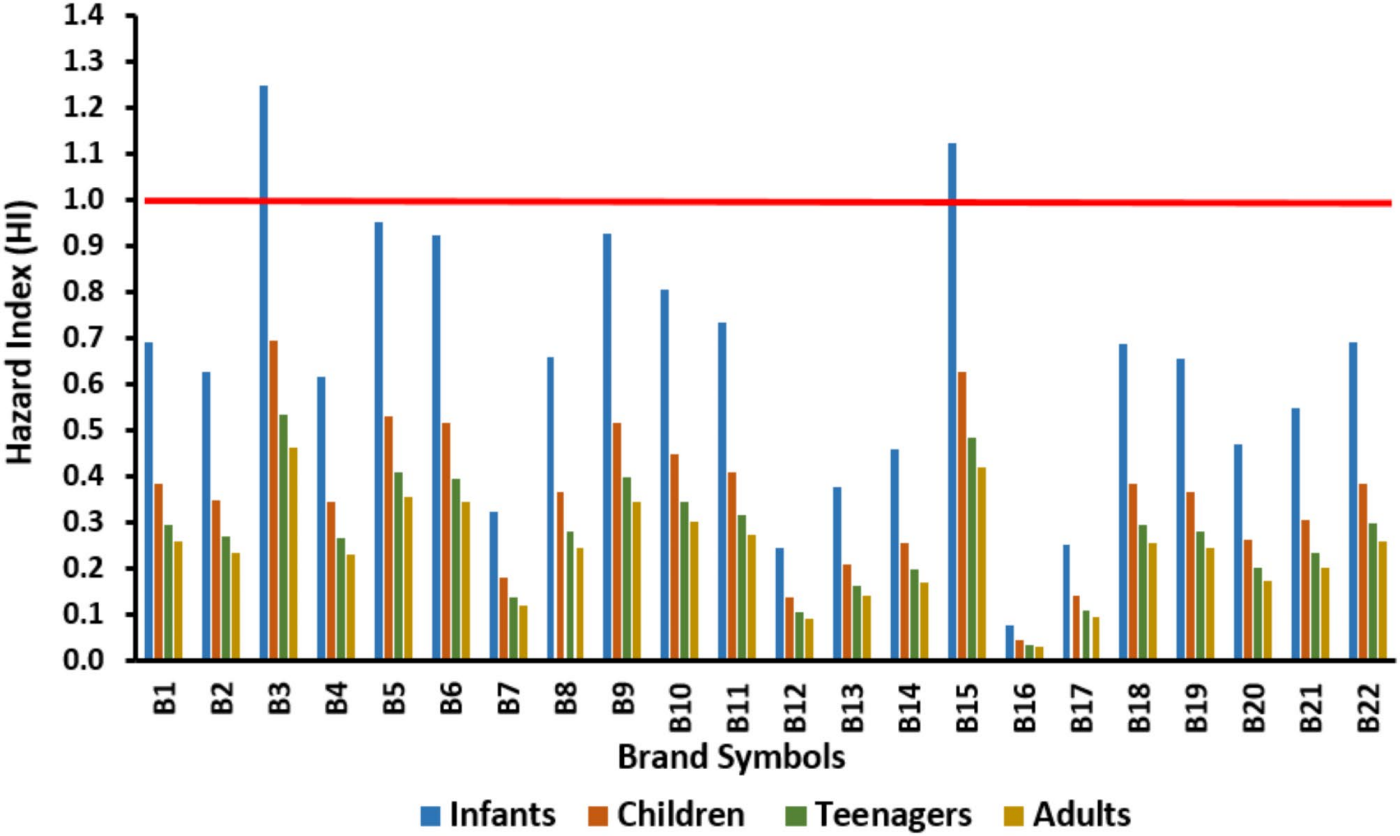
Hazard index value related to fluoride and nitrate concentration calculated for different bottled water brands and groups.

Hazard Index is used for sum of non- carcinogenic risk quotients of fluoride and nitrate. The HI values are presented in Fig. 3. HI ranges for infants, children, teenagers and adults varied from 0.08 to 1.25 (mean 0.64), 0.04 to 0.69 (mean 0.36), 0.03 to 0.53 (mean 0.27), and 0.03 to 0.46 (mean 0.24), respectively. From the finding (Figs. 3), 2 brands, namely, B3 and B15, exceed revealed HI > 1 for infants. For the other groups, HI values are less than 1, revealed no added risk related to sum of fluoride and nitrate non carcinogen risk for the studied bottled waters. In a comparison, HI values for all groups and the brands are as infants > children > teenagers > adults. Qasemi et, al. showed, HI for groundwater in 3.3%, 100%, and 100% of studied samples were more than 1 (HI > 1) for adults, children and infants, respectively, indicated non-carcinogen risks in infants, children, and adults through drinking water in most of the samples^54^. It has been reported that HI for sum fluoride and nitrate ranged from 0.02 to 7.66 (average of 2.96) in children and from 0.01 to 5.67 (average 2.19) in adults. Therefore, children were at higher risk compare to other groups^66^. Golaki et al. also reported the highest HI value for Kazerun children intaking nitrate, nitrite and fluoride from drinking water^56^.

### Monte Carlo simulation and sensitivity analysis

**Fig. 4:**
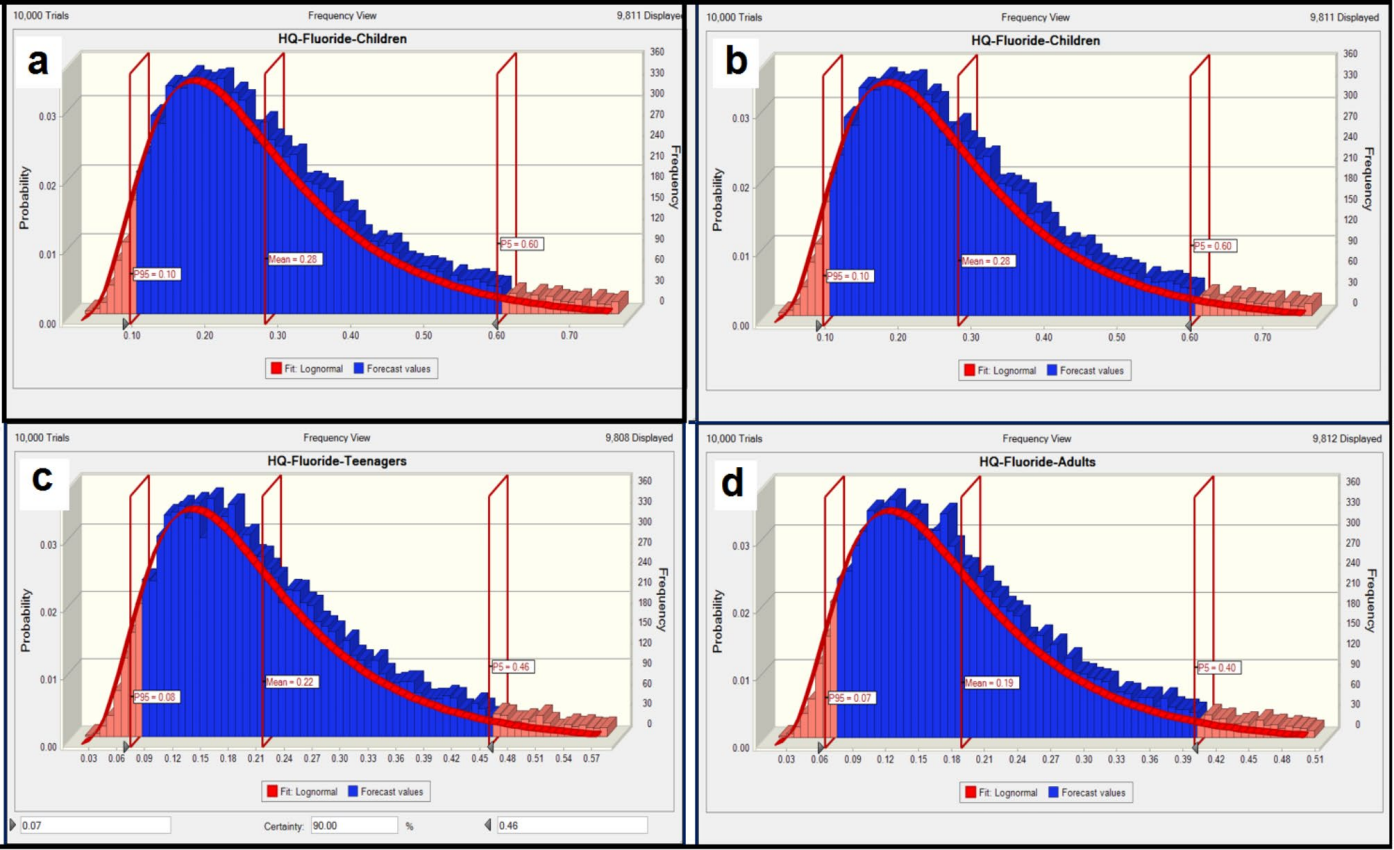
HQ value related to fluoride concentration calculated for different groups (a: infants, b: children, c: teenagers, c: adults).

**Fig. 5:**
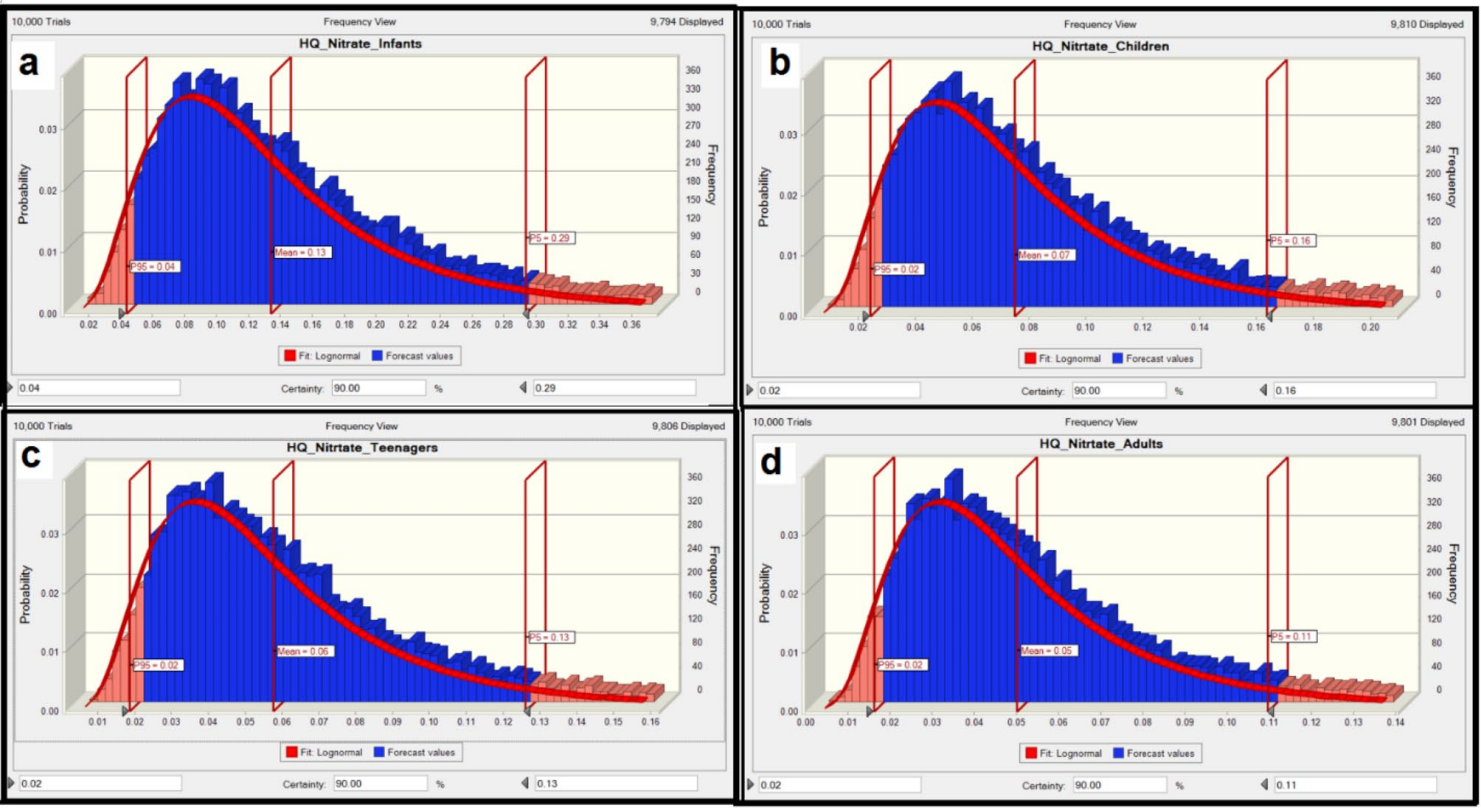
HQ value related to nitrate calculated for different groups (a: infants, b: children, c: teenagers, c: adults).

The Monte Carlo approach was applied to calculate uncertainties during risk assessment. Beside Monte Carlo simulations, other uncertainties were addressed especially the parameters that revealed by the sensitivity analysis. Fluoride concentration in samples collected from drinking bottled water in Kermanshah. To increase the validity of measurements, the experiments were conducted three times. Besides the application of single point estimation (calculated through Eqs. (1) and (2)), in this work, Monte Carlo simulation with 10,000 trials was conducted using Oracle Crystal Ball software to estimate the variance of HQ values. For this, the likelihood-based approach was utilized to evaluate nitrate and fluoride levels in the exposed groups. This method try to correct distribution of variables such as nitrate and fluoride concentration, ingestion rate (IR), and body weight (BW). The applicable values for infants, children, teenagers, and adults are presented in Table 1. The results are presented on the histograms in Fig. 4(a, b, c, and d). From the Fig. 4(a, b, c, and d), the fluoride-HQ values for the 95th percentile in infants, children, teenagers, and adults were 0.18, 0.1, 0.08, and 0.07 respectively. Although the values are significantly lower than 1, but comparatively, This amount is higher in infants than other groups, indicating that this age group is exposed to non-carcinogenic risks relating to nitrate, which is likely because of comparably less weight of his group^46,67^. In addition, histograms for simulating HQ results of nitrate in four exposed groups are displayed in Fig. 5(a, b, c, and d). According to Fig. 5, nitrate HQ for the 95th percentile in infant, children, teenager and adult age groups were 0.04, 0.02, 0.02 and 0.02, respectively, which indicates a non-carcinogenic risk for these groups. The highest 95th percentile of the calculated nitrate HQ was 0.04 for infants, compare to other groups. These results showed that HQ for nitrate for all groups are significantly lower than risk limit value (QH < 1) indicating negligible risk of developing non-carcinogenic effects, for the studies groups.

###  Sensitivity analysis

**Fig. 6:**
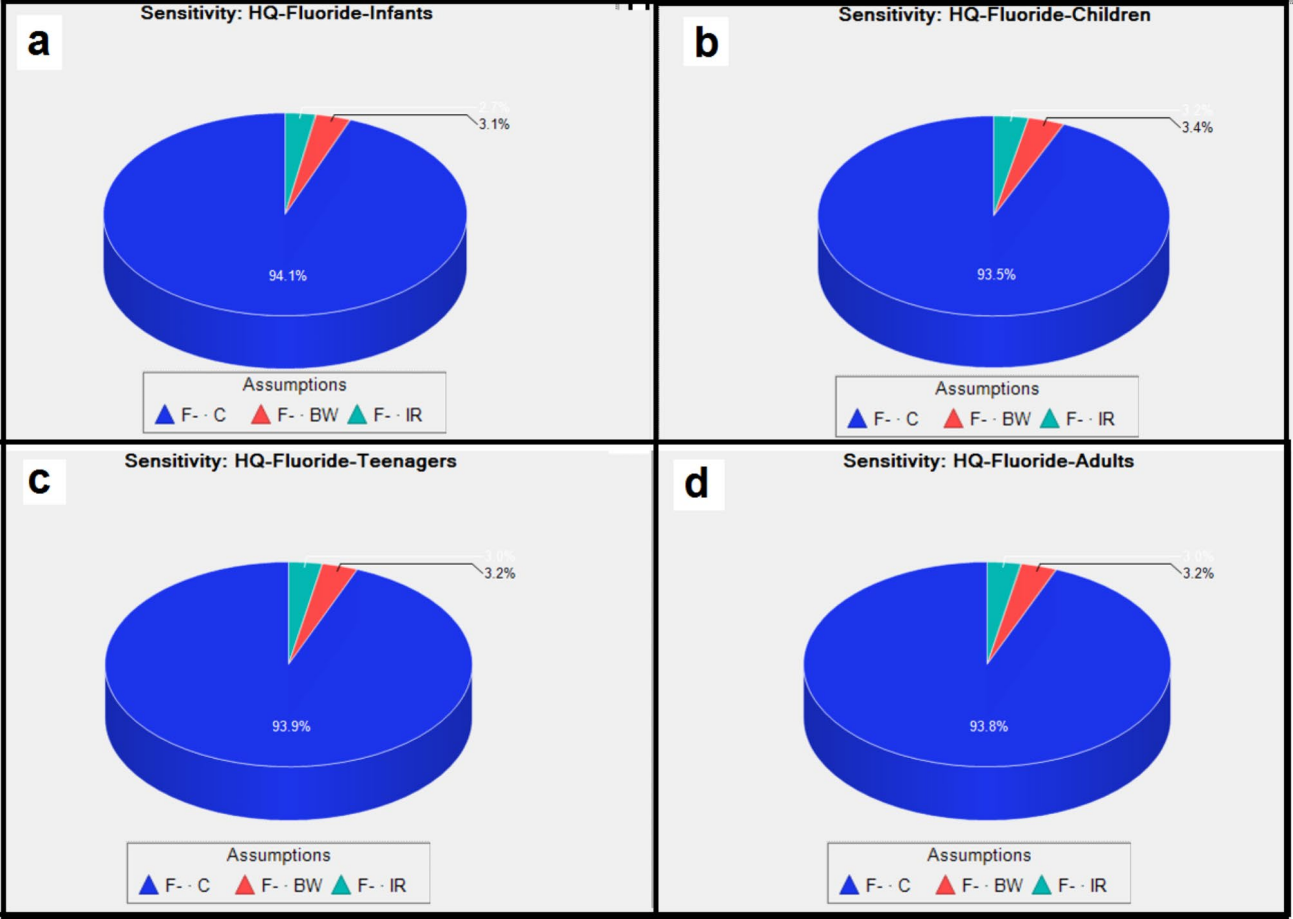
Sensitivity analysis of fluoride exposure for different 4 groups (a: infants, b: children, c: teenagers, d: adults).

Sensitivity analysis was carried out to pinpoint the most effective variables in HRA for the exposed groups to show uncertainty of the output response^29,62^. The results of sensitivity analysis for non-carcinogenic risk assessment in the different age groups (infants, children, teenagers, and adults) are depicted as pie charts in Fig. 6 (a, b, c, and d) for fluoride. For all the groups, among the influencing parameters (i.e. drinking water intake rate (IR), fluoride concentration (C), and exposure frequency (EF)), Fluoride concentration (C-F) showed the highest role for all the groups. Accordingly, the values of IR were 94.1%, 93.5%, 93.9%, and 93.8% for infants, children, teenagers, and adults respectively. These findings showed that fluoride concentration (C-fluoride) has a substantial impact on risk estimates for the four exposed groups. Moreover, compared to other variables, bw and IR have a less impact (≤ 3% for each variable) on the risk for exposure to fluoride for each group. Bazeli et al., Badeenezhad et al. and Revani et al. analysis showed the same finding for the crucial effect of fluoride concentration for same groups exposed^46,51^ while Ghahramani et al. revealed more effective role of IR than other factors for all the studied groups^29,50^.

**Fig. 7:**
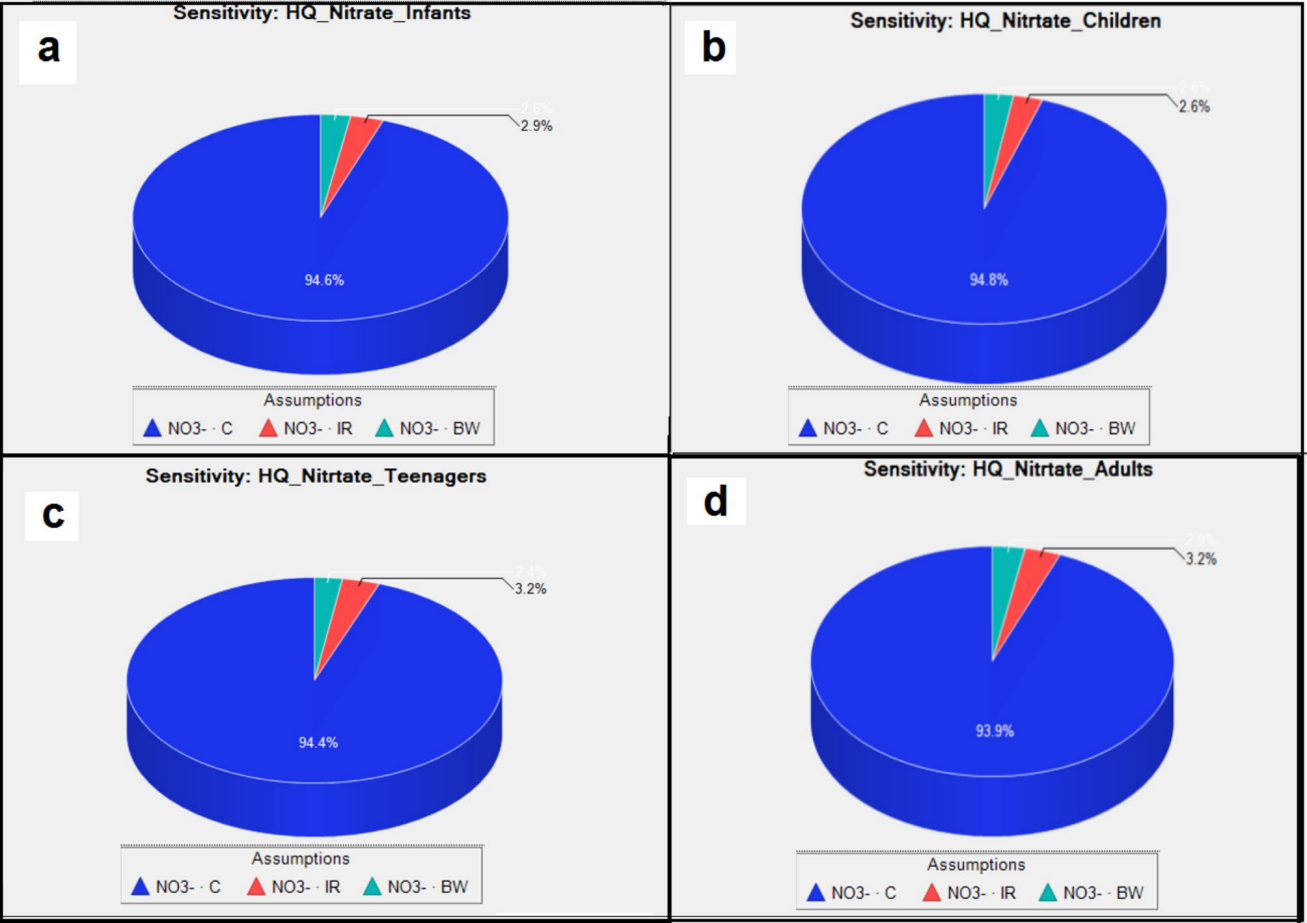
Sensitivity analysis of nitrate exposure for 4 different groups (a: infants, b: children, c: teenagers, d: adults).

The results of sensitivity analysis for non-carcinogenic risk assessment in the different age groups (infants, children, teenagers, and adults) are depicted as pie charts in Fig. 7 (a, b, c, and d) for nitrate. For all the groups, among the influencing parameters (i.e. drinking water intake rate (IR), fluoride concentration (C), and exposure frequency (EF)), Fluoride concentration (C-F) showed the highest role for all the groups. Accordingly, the values of IR were 94.6%, 94.8%, 94.4%, and 93.9% for infants, children, teenagers, and adults, respectively. These results showed that nitrate concentration (C-nitrate) has the most influence on risk estimates for all the exposed groups. In addition, compared to other factors, bw and IR have a less impact (≤ 3.2% for each variable) on the risk for exposure to nitrate for each group. Bazeli et al. and Badeenezhad et al. showed the same finding for the crucial effect of nitrate concentration for same groups exposed^46,50^. Therefore, the most crucial factor in the non-cancerous health risk due to nitrate and fluoride was ions content in bottled water. Therefore, reducing the concentration of nitrate and fluoride can lower the possibility of health risks.

## Conclusion

This study aimed to investigate nitrate and fluoride content in bottled water consumed in Kermanshah city and evaluate related health risks. In this study, fluoride and nitrate concentrations of all analyzed bottled waters were less than maximum WHO guideline and national Iranian standards. Among the different studied age groups, infants were proportionally at higher risk to health effects for both fluoride and nitrate exposure through bottled water. Although the risk of non-carcinogenic hazard in term of HQ was mostly less than 1 for fluoride exposure, the HQ values for only 2 brands of the bottled water were > 1 for infants group. In other hand, HQ was less than 1 for nitrate in all the age groups revealed non-carcinogen risks. However, the calculation of the HI, as an indication of sum effects of the two studied ions on the studied age groups, revealed that infants were proportionally at the higher risk compare to other groups. Estimated HI values for only 2 brands of bottled water were > 1 for infants group. Through Monte Carlo simulation showed that 95th Percentile for nitrate and fluoride exposure was less than 1 for all age groups, indicating a non-carcinogenic risk of nitrate and fluoride for 95% of the studied groups. Moreover, sensitivity analysis showered that concentration of fluoride and nitrate had the highest effect on HQ than other variables for all the groups. Finally, considering the other foods and beverages containing fluoride and nitrate can help the better estimating the risks and hazard index. However, continue monitoring of fluoride and nitrate levels in drinking waters should be conducted as possible preventive measures.

## Data Availability

All data has been provided in the manuscript.
